# The effect of an acute ingestion of Turkish coffee on reaction time and time trial performance

**DOI:** 10.1186/s12970-015-0098-3

**Published:** 2015-10-06

**Authors:** David D. Church, Jay R. Hoffman, Michael B. LaMonica, Joshua J. Riffe, Mattan W. Hoffman, Kayla M. Baker, Alyssa N. Varanoske, Adam J. Wells, David H. Fukuda, Jeffrey R. Stout

**Affiliations:** Institute of Exercise Physiology and Wellness, Sport and Exercise Science, University of Central Florida, 12494 University Blvd, Orlando, FL 32816-1250 USA

**Keywords:** Caffeine, Exercise, Cognition, Ergogenic effects

## Abstract

**Background:**

The purpose of this study was to examine the ergogenic benefits of Turkish coffee consumed an hour before exercise. In addition, metabolic, cardiovascular, and subjective measures of energy, focus and alertness were examined in healthy, recreationally active adults who were regular caffeine consumers (>200 mg per day).

**Methods:**

Twenty males (*n* = 10) and females (*n* = 10), age 24.1 ± 2.9 y; height 1.70 ± 0.09 m; body mass 73.0 ± 13.0 kg (mean ± SD), ingested both Turkish coffee [3 mg · kg^−1^ BW of caffeine, (TC)], and decaffeinated Turkish coffee (DC) in a double-blind, randomized, cross-over design. Performance measures included a 5 km time trial, upper and lower body reaction to visual stimuli, and multiple object tracking. Plasma caffeine concentrations, blood pressure (BP), heart rate and subjective measures of energy, focus and alertness were assessed at baseline (BL), 30-min following coffee ingestion (30+), prior to endurance exercise (PRE) and immediately-post 5 km (IP). Metabolic measures [VO_2_, V_*E*_, and respiratory exchange rate (RER)] were measured during the 5 km.

**Results:**

Plasma caffeine concentrations were significantly greater during TC (*p* < 0.001) at 30+, PRE, and IP compared to DC. Significantly higher energy levels were reported at 30+ and PRE for TC compared to DC. Upper body reaction performance (*p* = 0.023) and RER (*p* = 0.019) were significantly higher for TC (85.1 ± 11.6 “hits,” and 0.98 ± 0.05 respectively) compared to DC (81.2 ± 13.7 “hits,” and 0.96 ± 0.05, respectively). Although no significant differences (*p* = 0.192) were observed in 5 km run time, 12 of the 20 subjects ran faster (*p* = 0.012) during TC (1662 ± 252 s) compared to DC (1743 ± 296 s). Systolic BP was significantly elevated during TC in comparison to DC. No other differences (*p* > 0.05) were noted in any of the other performance or metabolic measures.

**Conclusions:**

Acute ingestion of TC resulted in a significant elevation in plasma caffeine concentrations within 30-min of consumption. TC ingestion resulted in significant performance benefits in reaction time and an increase in subjective feelings of energy in habitual caffeine users. No significant differences were noted in time for the 5 km between trials, however 60 % of the participants performed the 5 km faster during the TC trial and were deemed responders. When comparing TC to DC in responders only, significantly faster times were noted when consuming TC compared to DC. No significant benefits were noted in measures of cognitive function.

## Background

Coffee consumption has vast commercial, agricultural, and social importance, is pharmacologically active, and is often consumed for its stimulatory effects [1]. Coffee is a highly-concentrated source of caffeine (~2 %) [2–4]. The stimulatory effects of coffee are attributed to the pharmacological activity of caffeine, acting as an antagonist of adenosine receptors in the brain [5]. However, the wide variety of coffee species, roasting conditions, and extraction procedures employed results in a considerable amount of biological variance [1, 6, 7]. Unlike coffee traditionally consumed in the western world, Turkish coffee is not drip filtered, but rather its method of preparation involves slowly boiling water that is mixed with thin powdery grounds [7]. This style of preparation results in a greater amount of biologically active components remaining in the liquid, and likely contributes to the higher concentration of caffeine found in Turkish coffee compared to other coffee types and preparation styles [8].

The ingestion of caffeine has been reported to spare muscle glycogen, and increase fat oxidation through increased sympathetic nervous system activity [3, 9–11]. Several reviews examining the ergogenic benefits of caffeine use and endurance performance have concluded that caffeine use is positively related to greater performance in time trials and in exercise to exhaustion [10, 12, 13]. Caffeine anhydrous has long been thought to be superior for improving endurance performance as compared to coffee due to the presence of chlorogenic acid in coffee [3, 4, 14]. Recently, Hodgson et al. [4] compared the effects of decaffeinated coffee, regular coffee, caffeine anhydrous, and a placebo and demonstrated regular coffee to be as effective as anhydrous caffeine, when provided at the same relative dose, for improving endurance performance [4]. Differences in their study and others were partly attributed to the performance test used. Previous studies examining the ergogenic benefits of coffee have used prolonged submaximal runs [15], or using time to exhaustion tests [3, 9, 16], however Hodgson and colleagues [4] employed a time trial as their assessment. Considering that time to exhaustion tests have been shown to be highly variable from day to day [17], the use of time trials may provide a more reliable measure of endurance performance [18]. Therefore, we hypothesize that the ingestion of Turkish coffee will improve 5 km time trial performance, reaction time, and subjective measures energy, focus and alertness, but will not change metabolic or cardiovascular measures as compared to a decaffeinated placebo treatment.

## Methods

### Research design

Participants reported to the Human Performance Laboratory (HPL) for one familiarization session prior to experimental trials. During the familiarization session, participants were informed of all procedures and familiarized with all performance measures to reduce the possibility of a learning effect. Participants performed two experimental time trials with a minimum of 48 h between each trial. The average time between trials was 5.3 ± 2.7 days. During each trial, participants consumed either regular Turkish coffee (TC), or a decaffeinated Turkish coffee (DC) from the same manufacturer (Strauss Coffee, Lod, Israel).

**Fig. 1:**
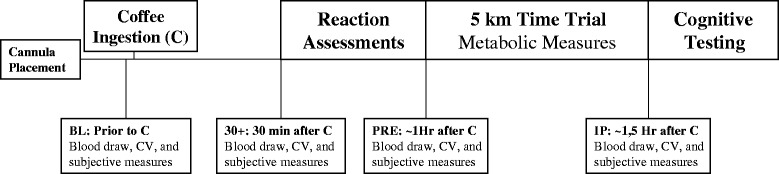
Study protocol

Participants reported to the HPL 2-h post-prandial and had not exercised for at least 24 h prior to each trial. In addition, participants were asked to not alter their dietary habits, but to replicate their dietary habits for the day before and day of each trial, and were requested to avoid caffeine consumption the day of the trial. Upon arrival to the laboratory participants were asked to lie comfortably in a supine position prior to the insertion of a cannula. Participants were then requested to sit comfortably for 15 min prior to the baseline (BL) blood draw. Following the BL blood draw participants were provided either the TC or DC. Additional blood draws occurred 30-min following coffee ingestion (30+), prior to the 5 km run (PRE), and immediately following the 5 km run (IP). Cardiovascular and subjective measures were also assessed at similar time points. Performance measures included reaction assessment, 5 km time trial and cognitive function. During the time trial oxygen consumption (VO_2_), minute ventilation (*V*_E_), respiratory exchange ratio (RER), and heart rate (HR) were assessed. The study protocol is depicted in Fig. 1.

### Participants

For inclusion in the study, participants were required to have a *V*O_2_peak greater than 35 ml∙kg^−1^∙min^−1^, and had to be regular caffeine and/or coffee consumers to increase homogeneity of the sample. Regular consumption was defined as ≥200 mg per day or ≥1 cup of coffee per day; the reported intake was 277 ± 183 mg · day^−1^. After initial testing, 23 recreationally-active individuals (men = 10, women = 13) between the ages of 18 and 35 were recruited to participate in this study. Two female participants were removed due to health reasons not associated with the study, and one for non-compliance. Therefore, data for 10 men (25.5 ± 1.8 y; 1.77 ± 0.05 m; 84.1 ± 7.1 kg; 48.0 ± 4.3 ml · kg · min^−1^) and 10 women (22.7 ± 2.0 y; 1.64 ± 0.07 m; 61.76 ± 5.28 kg; 42.0 ± 3.1 ml · kg · min^−1^) were included in the final analysis.

All participants completed a questionnaire to assess their ability to participate in physical activity and to ascertain any prior supplementation. Individuals who reported using substances that could potentially mask the effects of caffeine were excluded from the study. If participants were supplements that did not interfere with the effects of caffeine (e.g. protein), they were asked to continue their supplementation, and diet regiment so that the only difference between trials was the ingestion of TC or DC. Individuals self-reported to be free of musculoskeletal injury as determined by a PAR-Q. Participants were excluded if they had any history of cardiovascular disease, metabolic, renal, hepatic, or musculoskeletal disorders or were taking any other medication as determined by the medical history questionnaire. Following an explanation of all procedures, risks and benefits, each participant provided his/her informed consent to participate in the study. The Institutional Review Board of the University of Central Florida approved the research protocol.

### Treatment beverages

During each experimental trial, participants ingested either TC or DC 60-min prior to the 5 km time trial. Participants were provided 0.136 g coffee · kg^−1^ BW mixed in 200 ml of boiling water, resulting in 3 mg · kg^−1^ BW of caffeine per serving (e.g., for a participant with an average body weight of 70 kg, this was equivalent to 1.5 teaspoons of coffee powder). The Turkish coffee provided is marketed as Elite Turkish Coffee (Strauss Coffee, Lod, Israel) and the content provided was recommended by the manufacturer. There was no difference in appearance or taste between the TC and DC.

### Blood sampling

During each experimental trial, all blood samples were obtained using a 20-gauge Teflon cannula placed in a superficial forearm vein using a three-way stopcock with a male luer lock adapter. The cannula was maintained patent using an isotonic saline solution (Becton Dickinson, Franklin Lakes, NJ). Blood samples were collected at BL, 30+, PRE, and IP into a Vacutainer® tube, containing sodium heparin, and was kept chilled prior to each blood draw. Following collection samples were subsequently centrifuged at 4,000 × g for 15 min. The resulting plasma was placed into separate 1.8-mL microcentrifuge tubes and frozen at −80 °C for later analysis.

### Biochemical analysis

Of the 20 participants to complete the study 4 requested to not have blood drawn, thus a sample size of *n* = 16 was used for blood measures. Plasma glucose and lactate concentrations were determined using an automated analyzer (Analox GM7 enzymatic metabolite analyzer, Analox instruments USA, Lunenburg, MA). To eliminate inter-assay variance, all samples were analyzed in duplicate by a single technician. Coefficient of variation for each assay was 2.60 % for glucose and 0.79 % for lactate.

Plasma caffeine concentrations were quantified using high performance liquid chromatography (HPLC) with chromatographic conditions based upon previous work done in our lab [19]. Chromatography was performed on an Agilent Infinity 1260 HPLC (Agilent Technologies, Santa Clara, CA) consisting of a degasser, binary pump, auto-sampler, column thermostat, and photodiode array detector. A Zorbax Eclipse Plus C18 (4.6 × 150 mm, 5-μm) column and Zorbax analytical guard column (4.6 × 12.5 mm, 5-μm) were used for separation. Data were collected using OpenLAB chromatography data system, ChemStation edition.

All reagents were of HPLC grade. Caffeine, beta-hydroxyethyl-theophylline, sodium phosphate monobasic and sodium phosphate dibasic were purchased from Sigma-Aldrich (St. Louis, MO) to create the stock solution. Acetonitrile was purchased from Fisher Scientific (Pittsburgh, PA). HPLC grade water was prepared by reverse-osmosis and purified using a Milli-Q Direct 8 water purification system (EMD Millipore, Billerica, MA).

A 40 μg∙mL^−1^ stock solution of caffeine, theobromine and beta-hydroxyethyl-theophylline was prepared in water and sonicated. Twelve calibration standards were prepared from the stock solution in the range of 0.039 – 40 μg∙mL^−1^ by serial dilution of 1 mL of the stock solution. Beta-hydroxyethyl-theophylline (internal standard) working solution was prepared in water (10 μg∙mL^−1^).

An internal plasma sample was collected to serve as control and analyzed every 50 samples. Calibration standards, samples, and controls were prepared in the same fashion for linearity. 60 μL of the calibration standards or 50 μL of sample or quality control sample was added to 1.5 mL microcentrifuge tubes. 10 μL of the internal standard was subsequently added to the samples and controls, followed by 140 μL of chilled acetonitrile for deproteinization. Standards, samples, and controls were then vortexed vigorously for 30 s and placed in a refrigerator (4 °C) for two hours followed by centrifugation at 14,000 g for 15 min in a microcentrifuge to allow the protein to form a pellet. The supernatant (150 μL) was collected and subsequently transferred to a 0.45 μm polytetrafluoroethylene syringeless filter vial (GE Healthcare Mini-Uniprep™, Piscataway, NJ). A concentration of 300 μL of sodium phosphate buffer (mobile phase) was then added to the vial. The solution was filtered and injected into the HPLC using an auto-sampler.

The mobile phase consisted of 25 mM sodium phosphate (pH 7.0 ± 0.05 at 40 °C) and acetonitrile at a volume to volume ratio of 90:10. Buffer pH was achieved by mixing 5.41 g sodium phosphate monobasic anhydrous, and 7.80 g sodium phosphate dibasic anhydrous in 4 L of water at 40 °C. Buffer composition was calculated using Buffer Maker computer software (Marki, Poland) and verified using an Oakton pH 11 portable meter (Oakton Instruments, Vernon Hills, IL). Analysis was carried out under isocratic conditions via binary mixing of aqueous and organic phases at a flow rate of 1.5 mL∙min^−1^ under a system pressure of approximately 90 bars. Chromatograms were recorded at 275 nm with a run time of 6 min. Duplication of retention times for a known standard was used to verify column equilibrium prior to analysis.

### Cardiovascular measures

Heart rates and blood pressure (BP) were measured at each assessment time point using a wireless heart rate monitor (Polar® RS800CX, Kempele, Finland) and a mobile mercury sphygmomanometer (American Diagnostic Corporation Diagnostix™ 972, Hauppauge, New York).

### Subjective measures

Participants were instructed to assess their subjective feelings of energy, alertness, and focus using a 15-cm visual analog scale (VAS). The scale was anchored by the words “Lowest” and “Highest” to represent extreme ratings where the greater measured value represents the greater feeling. Questions were structured as “My level of energy is”, “My level of alertness is”, and “My level of focus is”. The validity and reliability of VAS in assessing subjective feelings have been previously established [20].

### Performance measures

#### Upper body reaction measurements

Measurement of upper body reaction time was performed on the Dynavision D2 Visuomotor Training Device (D2; Dynavision International LLC, West Chester, OH). The D2 is a light-training reaction device developed to train sensory motor integration through the visual system [21]. It consists of a board (4 ft x 4 ft) that can be raised or lowered relative to the height of the participant. It contains 64 target buttons (lights) arranged into five concentric circles surrounding a center screen that can be illuminated to serve as a stimulus for the participant. Participants were required to assume a comfortable athletic stance and stand at a distance from the board where they can easily reach all of the lights. The board height was kept consistent for all testing trials and was adjusted per participant so the center screen was located just below eye level. A total of three different reaction tests were conducted.

The first assessment measured the participant’s ability to react to a stimulus (light) as it changed position on the board. An initial stimulus (light) was present on the D2 in a random location. The stimulus (light) remained lit until it was touched by the participant. A stimulus (light) then appeared at another random location. The participant was instructed to successfully identify and touch as many stimuli (lights) as possible within 60 s. The number of successful “hits” was recorded for each trial. The ICC of this assessment has been reported to be 0.75 in our laboratory [21].

The second assessment was similar to the previous measure in that participants were also required to react to a visual stimulus (light) as it changed position on the board. However, during this trial the stimulus (light) remained lit for 1 s before it changed to another random location and the participant had to verbally recite a five digit number that was presented on the center screen of the D2 every 5 s. The appearance of the digits placed a cognitive demand on the information processing resources of the participant. The participant was instructed to successfully identify and touch each stimulus before it changed position and score as many touches as possible within 60 s. The number of successful “hits” was recorded for each trial. The ICC of this test has been reported to be 0.73 in our laboratory [21].

The third assessment measured the participant’s visual, motor, and physical reaction times to a visual stimulus with the dominant hand. The test was initiated when the participant placed and held his hand on an illuminated “home” button. At this point, a stimulus (light) was presented randomly in one of five locations, parallel to the home button. Visual reaction time was measured as the amount of time it takes to identify the stimulus (light) and initiate a reaction by taking their hand off the home button. Motor response time was measured as the amount of time it takes to physically touch the stimulus (light) with their hand following the initial visual reaction and was measured as the amount of time between the hand leaving the home button and touching the stimulus (light). Physical reaction time was measured as the total elapsed time from the introduction of the target stimulus (light) to the physical completion of the task (returning to the home button after touching the stimulus). All measures were recorded to the 1/100’s of a second. Participants performed this assessment ten times. The average time for all ten assessments was recorded. In our laboratory, the ICC of this test has been shown to be 0.84 (visual) and 0.63 (motor) [21].

#### Lower body reaction measurements

Lower body reaction time was measured using a 20-s reaction test on the Quick Board™ (The Quick Board, LLC, Memphis, TN) reaction timer. Participants stood on a board of five circles in a 2 x 1 x 2 pattern. Participants straddled the middle circle and reacted to a visual stimulus located on a display box that depicts one of five potential lights that corresponded with the circles on the board. Upon illumination of a light, the participant attempted to move the dominant foot to the circle that corresponds to the visual stimulus. Upon a successful “hit” with the foot, the next stimulus appeared. The total number of successful attempts during the 20-s test and the average time between the activation of the light and the response to the corresponding circle was recorded. The ICC of the Quick Board™ has consistently shown r > 0.90 in our laboratory [19, 22].

### Metabolic measures

#### Peak oxygen consumption (VO_2peak_) testing

During the familiarization trial an incremental test to volitional exhaustion was performed on a motorized treadmill (Woodway 4Front™, Waukesha, WI) to measure *V*O_*2*peak_. Open-circuit spirometry (TrueOne 2400® Metabolic Measurement System, Parvo Medics, Inc., Sandy, UT) was calibrated with room air and gases of known concentration, which was used to estimate VO_2_ (ml∙kg^−1^∙min^−1^) by sampling and analyzing breath-by-breath expired gases. *V*O_2peak_ was determined to be the highest 30-s *V*O_2_ value during the test and coincided with at least two of the following three criteria: (a) 90 % of age-predicted maximum heart rate; (b) respiratory exchange ratio > 1.1; and/or (c) a plateau of oxygen uptake (less than 150 mL · min^−1^ increase in *V*O_2_ during the last 60 s of the test). Previous work in our lab has shown the test-retest reliability for *V*O_2peak_ to be ICC = 0.96 (SEM 1.4 ml^.^kg^.^min^−1^) [23].

#### 5 km time trial measures

Prior to each 5 km time trial, open-circuit spirometry was calibrated with room air and gases of known concentration, which was used to estimate VO_2_ (ml∙kg^−1^∙min^−1^) by sampling and analyzing breath-by-breath expired gases. Expired gases—oxygen (O_2_), carbon dioxide (CO_2_), *V*_E_, RER, and heart rate were monitored continuously and expressed as 30-s averages. Ratings of perceived exertion (RPE) were recorded every 5-min during the time trial. The time needed to complete the 5 km was recorded to the nearest tenth of a second.

### Multiple object tracking and cognitive assessments

Subsequent to the removal of the intravenous catheter, after the IP blood draw, the participants were assessed for multiple object tracking using a Cave Automatic Virtual Environment (CAVE) system. The CAVE consists of a 7 ft × 7 ft × 7 ft room that includes a canvas projection screen on the front wall which served as the surface for image projection. During each session, the participant wore three dimensional glasses. A three-dimensional image of 8 tennis balls was projected onto the front screen. The participant tracked 4 of the 8 balls that moved in three-dimensions. At the beginning of each trial, the 8 balls appeared frozen on the screen for 2 s while half of them turned grey indicating the balls the participant was to track. After the 2 s, the balls all became the same color again and began to move in three dimensions. At the conclusion of the trial (8 s), the balls froze and a number appeared on each ball. The participant called out the numbers of the four balls they were supposed to be tracking. Velocity of movement began at a slow tracking speed and increased or decreased depending on whether the participant correctly identified the 4 correct balls. Each participant performed 20 trials per session. The velocity of movement that was most successful was recorded. Our laboratory has previously reported the ICC of this test to be 0.77 [24].

### Statistical analysis

Prior to statistical procedures, all data was assessed for normal distribution, homogeneity of variance, and sphericity. If assumption of sphericity were violated, a Greenhouse-Geisser correction was applied. Time trial and reaction time performance measures were analyzed with paired student’s *t* tests. Comparisons between trials were further analyzed using Cohen’s d. Cohen’s *d* values of 0.20, 0.50, and 0.80 were interpreted as small, medium, and large effect sizes, respectively [25]. All other measures were analyzed using a 2 x 4 [Trial (TC, DC) x Time (BL, +30, PRE, IP)] repeated measures analysis of variance (ANOVA). In the event of a significant interaction, LSD post-hoc tests were used for pairwise comparisons. Due to a significant difference at BL for the VAS energy response, a repeated measures analysis of covariance (ANCOVA) with BL measures serving as the covariate was used for this measure. For effect size, the partial eta squared statistic was calculated, and 0.01, 0.06, and 0.14 were interpreted as small, medium, and large effect sizes, respectively [26]. Additional analysis examined the effect of the trials in those participants that were determined to be responders. A responder was defined as a participant whose time for the 5 km time trial was faster during the TC trial compared to the DC trial. Significance was accepted at an alpha level of *p* ≤ 0.05. All data are reported as mean ± SD. Statistical analysis was performed using SPSS (version 21.0, SPSS Inc., Chicago, IL).

## Results

### Plasma caffeine concentrations

**Fig. 2:**
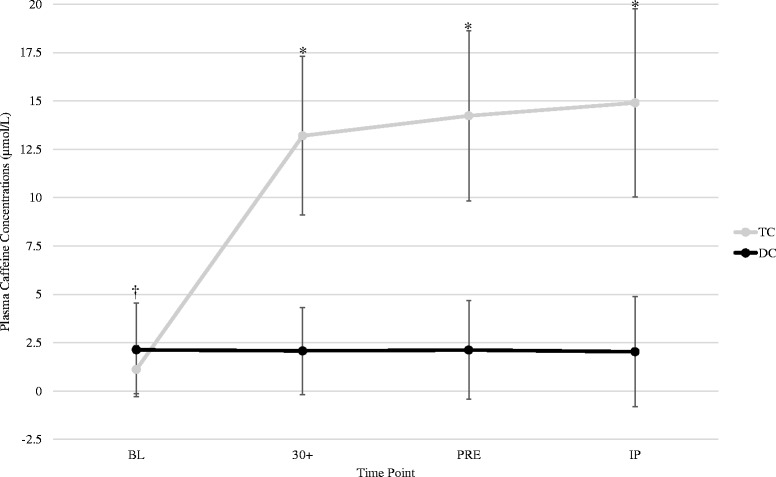
Plasma caffeine concentrations. * = Indicates significantly higher compared to DC. All values are reported as mean ± SD

Changes in plasma caffeine concentrations are displayed in Fig. 2. A significant interaction was observed between trials in plasma caffeine concentrations (F = 65.6, *p* < 0.001, η^2^ = 0.814). Significant elevations were observed at 30+, PRE and IP and each measure was significantly greater (*p* < 0.001) for TC than DC.

### Plasma glucose and lactate concentrations

No significant interactions were noted between trials in plasma glucose (F = 1.463, *p* = 0.245, η^2^ = 0.089) or lactate (F = 1.711, *p* = 0.211, η^2^ = 0.102). A main effect for time (F = 47.5, p < 0.001, η^2^ = 0.760) was seen for glucose. Significant elevations (*p* < 0.001) in plasma glucose were observed from BL (4.73 ± 0.87 mmol/L) to IP (7.74 ± 1.9718.5 mmol/L) for both groups. In addition, a significant main effect for time (F = 195.85, *p* < 0.001, η^2^ = 0.929) was observed in the plasma lactate response to exercise. Similarly, plasma lactate concentrations were significantly elevated (*p* < 0.001) from BL (2.29 ± 0.33 mmol/L) to IP (9.34 ± 0.49 mmol/L) for both groups.

### Blood pressure measures

**Fig. 3:**
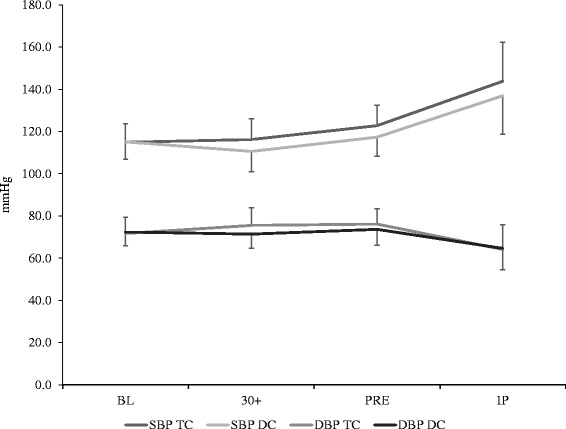
Blood pressure response during trials. All values are reported as mean ± SD

Blood pressure responses can be observed in Fig. 3. No significant interaction was noted between trials in systolic blood pressure (F = 1.121, *p* = 0.323, η^2^ = 0.056). When comparing groups across time, a significant main effect was observed (F = 65.7, *p* < 0.001, η2 = 0.776). Significant elevations (*p* < 0.001) in systolic blood pressure were observed from BL (115.0 ± 8.4 mmHg) to IP (140.4 ± 18.5 mmHg) in both groups. In addition, there was a significant main effect for trial (F = 6.089, *p* = 0.023, η^2^ = 0.243) indicating that systolic blood pressures were elevated during TC compared to DC when collapsed across all time points. Analysis of diastolic blood pressure responses revealed no significant interactions (F = 1.937, *p* = 0.134, η^2^ = 0.092). However, a significant main effect for time was noted (F = 13.32, *p* < 0.001, η^2^ = 0.412), indicating that when comparing trials across time, significant decreases (*p* = 0.008) were seen from BL (72.1 ± 7.1 mmHg) to IP (64.4 ± 10.8 mmHg). No other differences between groups were noted.

### Subjective measures of energy, alertness and focus

**Fig. 4:**
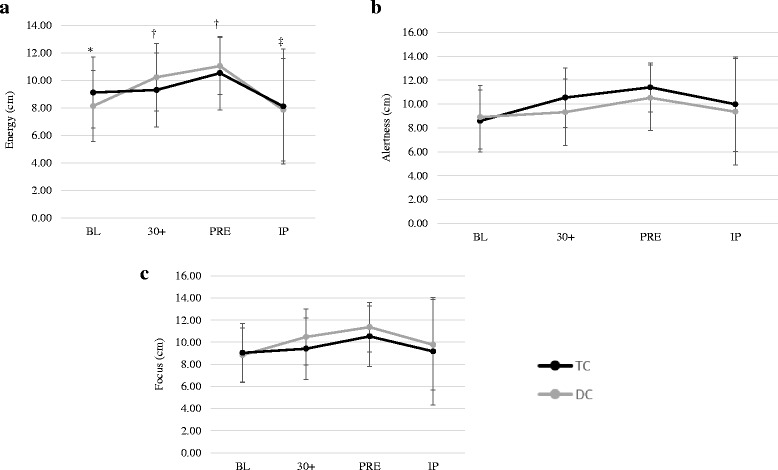
Subjective measures of energy, alertness, and focus.^*^ = Indicates significantly different at baseline;^†^ = Indicates significant interaction effect with TC higher than DC at given time point;^‡^ = Indicates significant interaction effect with DC higher than TC;^$^ = Indicates significantly lower than 30+ and PRE;^§^ = Indicates significantly lower than PRE. Enervy values adjusted for the initial differences in BL energy. All values are reported as mean ± SD. **a** = Energy; **b** = Alertness; **c** = Focus

Changes in subjective measures can be seen in Fig. 4. A significant interaction was noted between trials for subjective measures of energy (Fig. 4a) (F = 7.637, *p* = 0.007, η^2^ = 0.310). After adjusting for BL differences the adjusted group means were higher during TC at 30+ and PRE compared to DC, and lower during TC at IP compared to DC. No significant interaction (Fig. 4b; F = 2.231, *p* = 0.122, η^2^ = 0.105) was observed between trials in alertness. However, analysis of the main effect for trial indicated a trend (F = 3.775, *p* = 0.067, η^2^ = 0.166) between groups across time, suggesting that alertness during TC (10.12 ± 0.50) tended to be higher than DC (9.52 ± 0.58). No significant interaction (Fig. 4c; F = 1.320, *p* = 0.277, η^2^ = 0.065) were noted between trials in focus. However, analysis of the main effect for trial indicated a trend (F = 3.189, *p* = 0.090, η^2^ = 0.144) between groups across time, suggesting that focus tended to be higher during TC (10.16 ± 0.50) compared to DC (9.54 ± 0.61).

### Reaction measures

The average number of “hits” in 60 s was significantly greater for TC (85.1 ± 11.7) compared to DC (82.0 ± 13.7) (*p* = 0.023, *d* = 0.57). No significant differences between TC and DC were observed for visual (0.31 ± 0.06 s versus 0.30 ± 0.03 s, respectively) (*p* = 0.557, *d* = 0.14), motor (0.14 ± 0.06 s versus 0.15 ± 0.06 s, respectively) (*p* = 0.269, *d* = 0.26), physical (0.49 ± 0.10 s versus 0.49 ± 0.04 s, respectively) (*p* = 0.610, *d* = 0.12), or lower body reaction time (24.3 ± 4.6 hits versus 25.3 ± 4.4 hits, respectively) (*p* = 0.263, *d* = 0.26).

### Time trial performance

No significant difference (*p* = 0.192, *d* = 0.30) was observed between TC (1685.0 ± 216.6 s) and DC (1717.2 ± 255.7 s) in time to complete the 5 km time trial. However, average RER during the time trials was significantly greater for TC (0.98 ± 0.05) compared to DC (0.96 ± 0.05) (*p* = 0.019, *d* = 0.57). No significant differences between TC and DC were observed for average HR (174.5 ± 8.7 bpm and 175.2 ± 9.0 bpm, respectively) (*p* = 0.623, d = 0.11), average VO_2_ (35.63 ± 4.20 ml · min · kg^−1^ and 35.23 ± 4.70 ml · min · kg^−1^, respectively) (*p* = 0.415, d = 0.19), average *V*_E_ (77.1 ± 13.2 L∙min^−1^ and 71.9 ± 17.8 L∙min^−1^, respectively) (*p* = 0.099, d = 0.39) and average RPE (13.4 ± 1.5 and 13.5 ± 1.9, respectively) (*p* = 0.774, d = 0.06).

**Fig. 5:**
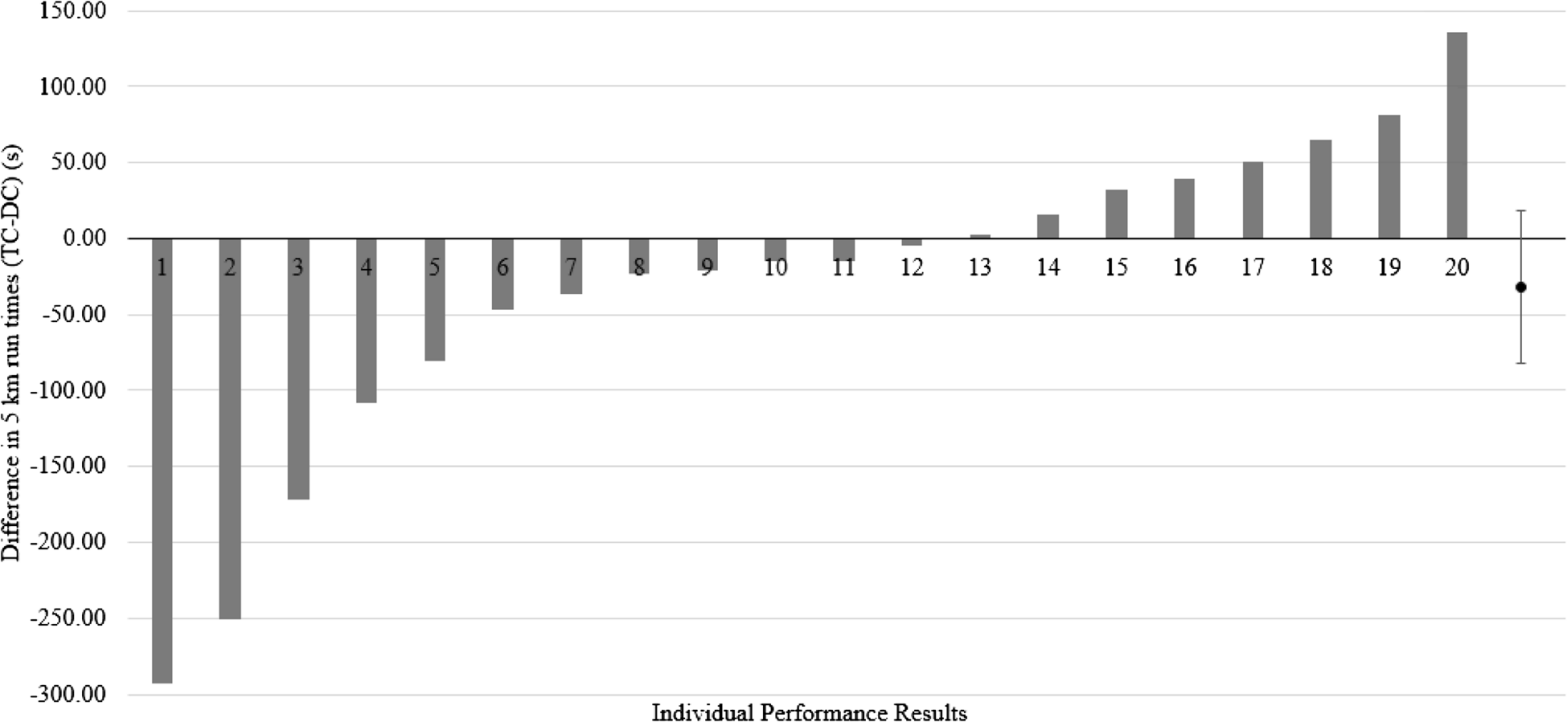
Individual 5 km times

When examining individual performance results in the 5 km time trial (see Fig. 5) it was observed that 12 of the 20 participants (6 males and 6 females) ran the 5 km faster when consuming TC than when consuming DC. When comparing performance differences in these 12 responders, a 5.0 % difference (*p* = 0.009) was seen in time for the 5 km time trial between TC (1685 ± 249 s) and DC (1774 ± 283 s).

### Cognitive measures

No significant differences between TC and DC were observed for multiple object tracking (1.29 ± 0.68 AU versus 1.35 ± 0.53 AU, respectively) (*p* = 0.643, *d* = 0.11).

## Discussion

The present study is the first investigation to examine the ergogenic benefits and metabolic responses of Turkish coffee. The results of this study indicated that plasma caffeine concentrations were significantly elevated within 30 min following TC ingestion. Participants consuming TC experienced significantly greater reaction performance and subjective feelings of energy. Although no significant differences were noted in time trial performance, during the TC time trial participants experienced a significantly higher average RER than that seen during the DC trial, suggesting a higher utilization of carbohydrate to meet metabolic demands. Our results are not consistent with previous investigations which have shown either a decrease or no effect of caffeine on RER [3, 9, 27, 28]. Although speculative, differences in the exercise protocol utilized in this study may have contributed to these contrasting results. In the present study a time trial protocol was used, while other studies employed a time to exhaustion protocol. The goal of a time trial is to complete the distance in as fast a time as possible, whereas a time to exhaustion protocol requires the participant to exercise at a predetermined pace. As such, if the goal is to complete a given distance as fast as possible the RER would be greater than that seen during a set-paced trial. Previous research has reported that coffee ingestion can increase average power output during a time trial [4]. In addition, caffeine is a known central nervous system stimulator acting as an adenosine antagonist, and subsequently increasing sympathetic nervous system activity [4, 11]. Although no significant differences were noted in time trial performance, the greater RER response, and a trend observed in the *V*_E_ response during the TC trial, does suggest a greater anaerobic effort during the run. This is also consistent with the greater perceived energy reported by participants during TC compared to DC. Interestingly, 60 % of the subjects (12/20) ran faster during the 5 km time trial during TC compared to DC. When comparisons between TC and DC were performed in the responders only, the time differentials between trials were significantly different. Although speculative, the 60 % responder rate is similar to the 61.1 % frequency observed in habitual coffee drinkers possessing a gene associated with enhanced cytochrome P450 1A2 [29]. Cytochrome P450 is a liver enzyme that is involved with caffeine metabolism [30]. Thus, those individuals with this polymorphism would potentially have a faster metabolism of caffeine, and potentially have a greater ergogenic response [29, 30].

Coffee and caffeine have been associated with enhancing the ability to perform mental tasks, including enhanced alertness and attention during activities of daily living and physical activity [19, 31–33]. Results of this study showed significant increases in subjective measures of energy, with trends demonstrated in alertness, and focus during the TC trial. Greater feelings of energy at 30+ and PRE corresponded to the significant elevations in plasma caffeine concentrations. These results are in agreement with other investigations showing that a 300–600 mg dose of caffeine can provide beneficial effects on measures of sleepiness, vigor, fatigue, and attentiveness [19, 31, 34–36]. The lower feelings of energy in conjunction with a 7 % increase in *V*_E_, and greater carbohydrate utilization in TC compared to DC likely reflects a higher running velocity used during this trial. The differences in subjective feelings of energy at IP were more difficult to explain and may be spurious.

In the current study, average upper body reaction time, measured as the number of successful “hits” in 60 s, was significantly improved for the TC trial compared to DC. This is consistent with previous studies that have observed improved reaction performance with caffeine ingestion [35, 36]. Although improvements were not noted in all reaction drills, it is likely that the testing duration of the visual, motor and physical upper body reaction test, and the lower body quickness assessment was not sufficient to yield significant changes. Additionally no significant effects were noted in any of the cognitive function assessments. Although caffeine is known to have positive effects on simple reaction time and visuo-spatial reasoning in a dose-dependent manner [34], the effects of caffeine on cognition is not as well supported [12, 21]. The present study enrolled participants who were regular consumers of caffeine. Chronic ingestion of caffeine (3 mg · kg^−1^ BW) has been shown to have no beneficial effects on a four-choice reaction time test, or on measures of alertness and attention [37]. The present study may not have provided enough caffeine for habitual users to demonstrate beneficial effects on attention, which may have affected their performance on tests of cognitive abilities.

Circulating plasma glucose and lactate were elevated following exercise, but no differences were noted between trials. This is consistent with some studies [3, 27, 28, 38], but in contrast to others [4]. Hodgson and colleagues [4] reported significant increases in plasma glucose concentrations at the end of a 30-min steady state cycling protocol following caffeinated coffee ingestion (5 mg caffeine · kg^−1^ BW). Studies that did not see any difference in the glucose or lactate response, including the present study, provided equivalent caffeine doses ranging from 3.0 mg - 4.5 mg caffeine · kg^−1^ BW dose [3, 39]. It appears that caffeine doses of 5 mg · kg^−1^ or greater appear necessary to significantly elevate plasma glucose.

The time course of caffeine elevation following the consumption of Turkish coffee seen in this study was similar to that seen following the ingestion of instant coffee [4], and similar to other investigations investigating caffeine pharmacokinetics [3, 10, 28]. Acute and chronic ingestion of coffee or anhydrous caffeine has been shown to cause an increase in sympathetic nervous system activity and subsequent increase in systolic blood pressure [11, 40]. Although no significant interactions were noted between the trials in the systolic blood pressure response, when collapsed across time systolic blood pressures were significantly elevated for TC compared to DC, consistent with the known physiological response following caffeinated coffee ingestion [41].

## Conclusions

Acute ingestion of 3.0 caffeine · kg^−1^ BW of Turkish coffee results in a significant elevation in plasma caffeine concentrations within 30 min of consumption. Elevations in plasma caffeine did appear to result in significant performance benefits in reaction time during a 60-s visual response stimulus and increased subjective feelings of energy in habitual caffeine users. Although no significant effects were noted in time trial performance during a 5 km run, 60 % of the participants were deemed responders and performed significantly faster (5 %) in the 5 km when consuming Turkish coffee compared to the decaffeinated trial. No significant benefits though were noted in measures of cognitive function. Considering the paucity of research on performance and Turkish coffee consumption, additional research examining the ergogenic benefits of pre-exercise ingestion appears warranted.
